# Man versus machine? Acquired long QT syndrome in a patient with anorexia nervosa

**DOI:** 10.1111/anec.12704

**Published:** 2019-09-24

**Authors:** Tomio Tran, Michael Brunnquell, Philip S. Mehler, Mori J. Krantz

**Affiliations:** ^1^ Internal Medicine Residency University of Colorado Aurora Colorado; ^2^ Hospital Medicine Denver Health Medical Center Denver Colorado; ^3^ University of Colorado School of Medicine Aurora Colorado; ^4^ Eating Recovery Center ACUTE at Denver Health Denver Colorado; ^5^ University of Colorado School of Medicine Aurora Colorado; ^6^ Division of Cardiology School of Medicine University of Colorado Aurora Colorado; ^7^ Division of Cardiology Denver Health Medical Center Denver Colorado

**Keywords:** anorexia nervosa, computerized, hypokalemia, manual, QTc prolongation, torsade de pointes

## Abstract

Computer‐generated Bazett‐corrected QT (QTcB) algorithms are common in clinical practice and can rapidly identify repolarization abnormalities, but accuracy is variable. This report highlights marked rate‐corrected QT (QTc) interval prolongation not detected by the computer algorithm. A 26‐year‐old woman with anorexia nervosa was admitted with severe hypokalemia and ventricular ectopy. Computer‐generated QTcB was 485 ms, while manual adjudication yielded a QTcB of 657 ms and a Fridericia‐corrected QT (QTcF) interval of 626 ms using digital calipers. Computer‐generated QTc intervals may aid in clinical decision‐making. However, accuracy is variable, particularly in the setting of ectopy, and requires manual verification.

## 1 CASE DESCRIPTION

A 26‐year‐old woman with history of borderline personality disorder and severe anorexia nervosa, binge–purge subtype, was admitted for medical stabilization prior to enrollment in a residential eating disorder program. She has had multiple admissions for medically supervised electrolyte repletion and refeeding.

**Figure 1:**
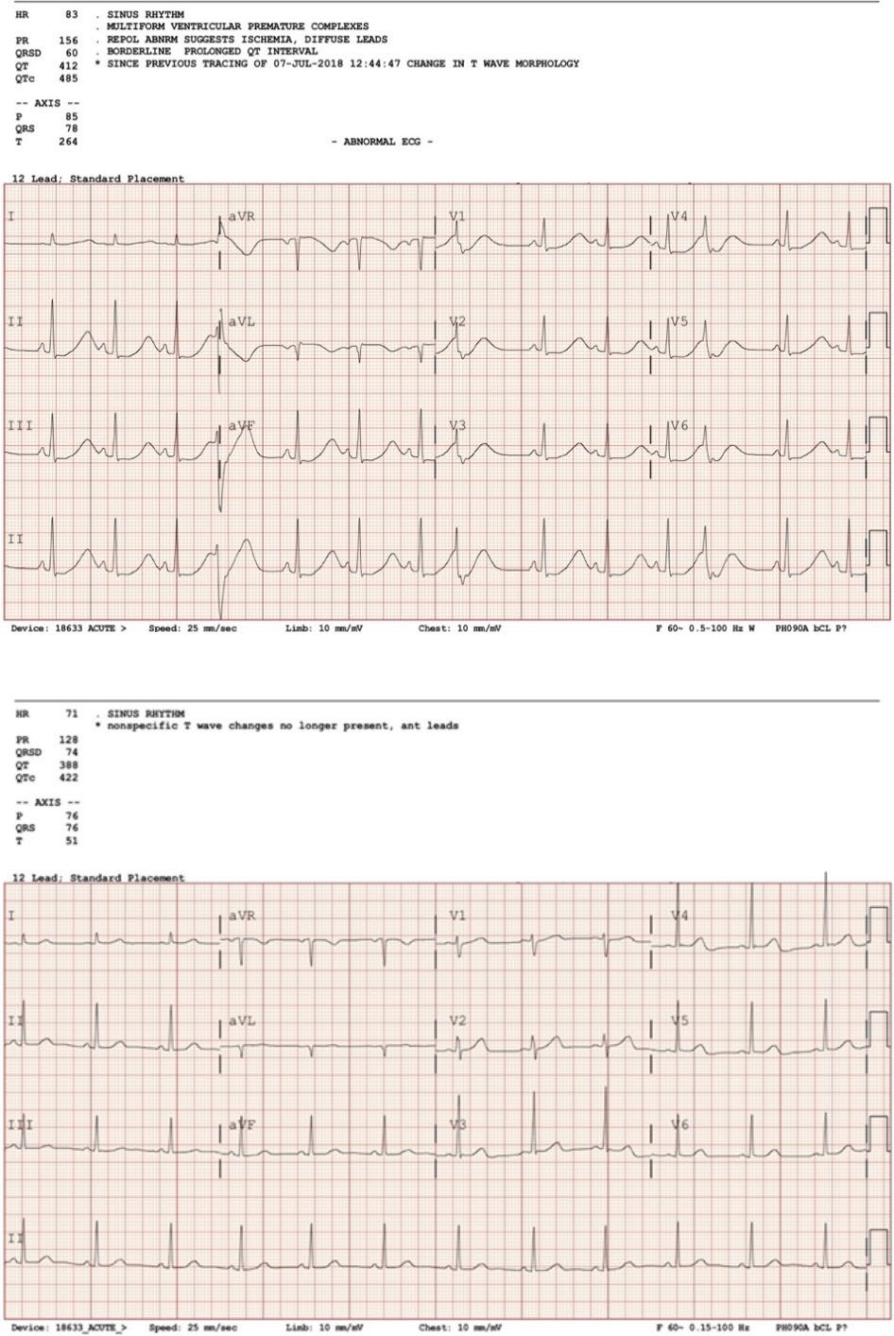
QTc prolongation underestimated in the setting of profound hypokalemia with ventricular ectopy (top panel) and QTc interval normalized after potassium repletion (lower panel)

Upon admission, her weight was 44.6 kg, height was 165.1 cm, body mass index was 13.04 kg/m^2^, and ideal body weight percentage was 62.4%. Her initial vitals showed hypotension with a blood pressure of 85/60 mm Hg and physical exam showed cachexia, temporal wasting, and an irregularly irregular heart rhythm. Admission laboratory values showed severe hyponatremia, hypokalemia, hypochloremia, and a metabolic alkalosis (Table 1 **)**. Initial 12‐lead electrocardiography (ECG) showed sinus rhythm with frequent, multifocal, VPCs (ventricular premature complexes) and “R‐on‐T” phenomenon (Figure 1, top panel).

**Table 1:** Serum Electrolyte Values

Laboratory value	Admission	Day 2	Discharge
Sodium (135–143 mmoL/L)	123	131	142
Potassium (3.6–5.1 mmoL/L)	1.3	3.2	3.9
Chloride (99–110 mmoL/L)	60	83	109
Bicarbonate (18–27 mmoL/L)	56	41	23
Creatinine (0.5–1.39 mg/dl)	1.3	1.1	0.76
Magnesium (1.3–2.2 mEq/L)	1.9	2.1	2.0

Given the presence of severe hypokalemia and high risk for torsade de pointes, the patient was transferred to the intensive care unit for cardiac monitoring and aggressive electrolyte repletion. Medications generally used for gastrointestinal symptoms associated with anorexia nervosa were avoided due to their QT‐prolonging properties. The patient received gentle fluid resuscitation with normal saline at 50 cc/hour and 170 mEq of potassium by oral and intravenous routes over a 36‐hr period. Serum potassium rapidly normalized, and the metabolic alkalosis resolved (Table 1). Repeat ECG showed resolution of marked QTc prolongation and ventricular ectopy (Figure 1, lower panel).

## 2 DISCUSSION

This patient exhibited T‐wave broadening, ST segment depressions, and QTc interval prolongation which are typical of hypokalemia (El‐Sherif & Turitto, 2011). In addition, the patient also displayed frequent multifocal VPCs with “R‐on‐T phenomenon,” which is a harbinger for the initiation of polymorphic ventricular tachycardia; torsade de pointes. The commercial algorithm calculated the QTc at 485 ms; however, manual calculation yielded a Bazett QTc interval of 657 ms and a Fridericia QTc interval of 626 ms. There are a number of explanations for the discrepancy between the algorithm and manual calculations. One reason is that there is no gold standard for computer measurements of the QTc interval, resulting in the use of a variety of commercial algorithms, which have shown small, but statistically significant differences (Kligfield et al., 2018). Also, measurement of the QTc interval is contingent upon the algorithm's ability to accurately identify the end of the T wave (T‐wave offset). We previously reported that within the context of a thorough QTc study, a computer‐assisted manual method was more precise than a commercially available automated method (Barbey, Connolly, Beaty, & Krantz, 2016), indicating that human inspection of ECGs can improve diagnostic performance, which is particularly critical in outlier analysis.

One inherent limitation of computer algorithms is the lack of contextual framework regarding patient's status, which may influence how a clinician interprets an ECG. In a retrospective study comparing the accuracy of computer interpretations confirmed by electrophysiology subspecialists, primary cardiologists directly caring for a patient performed better than the cardiologists overreading the computer interpretation (Anh, Krishnan, & Bogun, 2006), which suggests that knowledge of a patient's clinical course influences ECG interpretation and diagnostic accuracy. In this case, the clinician's knowledge about the patient's ectopy, abnormal electrolytes, and diagnosis of anorexia nervosa, which may be associated with QTc prolongation (Sachs, Harnke, Mehler, & Krantz, 2016), prompted vigilance in manually adjudicating the QTc interval and escalating the level of care.

Clinicians often rely on computer algorithms for QTc interval measurements. It is informative to review how automated algorithms adjudicate the QTc interval and their potential pitfalls. Generally, computer algorithms identify a normal QRS complex and the intervals are calculated from this landmark. These intervals are then averaged to create a global measurement. In patients with hypokalemia, the broadening of T waves and prominent U waves may create challenges in the recognition of fiducial points for calculating the QTc interval. Moreover, in this case, frequent ventricular ectopy occurred before termination of the preceding T wave, which leads to underestimation of the QTc interval by the algorithm. When all intervals were averaged, this leads to a globally shortened calculated QTc interval. Furthermore, it has been shown that the QTc interval after ventricular ectopy is not reliable (Reiffel & Reiffel, 2009) in creating a global assessment. Overall, these data imply that as an ECG manifests progressively more abnormalities, fiducial point discernment becomes correspondingly limited, therefore the accuracy of computer algorithms becomes correspondingly limited.

Among patients with eating disorders, QTc interval prolongation has been variably described. We previously found that QTc prolongation is most likely not an intrinsic feature of the disease itself, but generally reflects extrinsic factors such as hypokalemia and medications that inhibit the delayed rectifier potassium channel when using a computer‐assisted manual digital method (Krantz et al., 2012). Standard of care treatment of eating disorders includes numerous agents to treat gastrointestinal symptoms, including refeeding, but each can prolong the QTc interval. Common medication examples include ondansetron for nausea, as well as metoclopramide and azithromycin for gastroparesis (Mehler, 2001). Without manual assessment of the QTc interval, treatment of gastrointestinal symptoms could have further prolonged the QTc interval and increased this patient's risk of developing malignant ventricular arrhythmia. This case therefore suggests that in the battle between human versus machine, human oversight continues to have value both in research (Barbey et al., 2016) and within clinical arenas.

## CONFLICTS OF INTEREST

The authors declare that they have no conflicts of interest.
